# Letter to the Editor ‘Metabolic syndrome and surgical complications: a systematic review and meta-analysis of 13 million individuals’

**DOI:** 10.1097/JS9.0000000000001640

**Published:** 2024-05-15

**Authors:** Biao Zhang, Qihang Yuan, Dong Shang

**Affiliations:** a Pancreas and Biliary Center, Department of General Surgery, Clinical Laboratory of Integrative Medicine, The First Affiliated Hospital of Dalian Medical University; b Institute (College) of Integrative Medicine, Dalian Medical University, Dalian, Liaoning, People’s Republic of China

*Dear Editor,*

We recently carefully read the article titled ‘Metabolic syndrome and surgical complications: a systematic review and meta-analysis of 13 million individuals’ published by Norris *et al*.^1^ in the *International Journal of Surgery*. We commend Norris and colleagues for their valuable contribution to the field by conducting a systematic review and meta-analysis of the association between metabolic syndrome and surgical complications. This is a very valuable study that provides key evidence and guidance for the perioperative management of patients with metabolic syndrome. However, there are still some issues in this study that deserve further discussion and clarification.

A total of 61 articles were included in this study, including 1 919 347 patients with metabolic syndrome and 11 248 114 patients without metabolic syndrome. The large number of patients increases the reliability and generalizability of the results. However, we believe some aspects warrant further discussion and clarification. First, it is essential to address the potential overlap of patients across studies. Simply removing duplicate articles may not suffice, as different studies may include overlapping patient populations. Therefore, a thorough examination of patient sources in each included study is necessary to eliminate duplicate patients and enhance the accuracy of the results. Second, these patients come from various countries and regions (including Europe and the United States, Asia, and South Africa) and encompass different ethnic groups. There are variations in physical constitution among different ethnicities and disparities in medical resources and standards across different countries and regions. Therefore, to make the research results more reasonably applicable to patient management in various regions, it is very necessary to conduct subgroup analysis for different regions or different ethnic groups.

This study suggested that metabolic syndrome significantly increases the risk of surgical complications. However, the types of surgery include traditional open surgery and minimally invasive surgery, as well as surgery on different parts and organs, and can be divided into different levels according to the risk and complexity of the surgery. The risk of postoperative complications varies with different types of surgery. A more nuanced grouping of surgical types is worthwhile to provide more tailored guidance for the perioperative management of patients with metabolic syndrome.

The substantial heterogeneity observed in the meta-analysis (30-day mortality, *I* ^2^=93%; 30-day cardiovascular complications, *I* ^2^=68%; Surgical site infections, *I* ^2^=71%; Hospital length of stay, *I* ^2^=98%; Readmission, *I* ^2^=86%) undermines the credibility of the results. It is recommended that the authors utilize appropriate subgroup analyses, sensitivity analyses, or meta-regression to identify and address the sources of heterogeneity and thereby improve the stability and reliability of the meta-analysis results^2^.

Overall, we applaud the authors for their important contribution to the field, providing a meaningful synthesis of evidence. At the same time, we also look forward to further discussion to enhance our comprehensive understanding and provide high-quality, evidence-based guidance for the perioperative management of patients with metabolic syndrome.

## Ethical approval

Not applicable.

## Consent

Not applicable.

## Sources of funding

Not applicable.

## Author contribution

B.Z. and D.S.: conceptualized the manuscript; B.Z., Q.Y., and D.S.: wrote and revised the manuscript.

## Conflicts of interest disclosure

There are no competing financial interests listed by the authors.

## Research registration unique identifying number (UIN)

Not applicable.

## Guarantor

Biao Zhang, Qihang Yuan, and Dong Shang.

## Data availability statement

Not applicable.

## Provenance and peer review

Although our work was not by invitation, we believe that our findings will be helpful in improving the current work. Active discussion and exploration help to provide high-quality, evidence-based guidance for the perioperative management of patients with metabolic disease syndrome.
